# Evaluation of Brain Targeting and Antipsychotic Activity of Nasally Administrated Ziprasidone Lipid–Polymer Hybrid Nanocarriers

**DOI:** 10.3390/ph16060886

**Published:** 2023-06-15

**Authors:** Hadel A. Abo El-Enin, Alaa S. Tulbah, Hany W. Darwish, Rania Salama, Ibrahim A. Naguib, Heba A. Yassin, Hend Mohamed Abdel-Bar

**Affiliations:** 1 Department of Pharmaceutics, National Organization of Drug Control and Research (NODCAR) (Previously), Egyptian Drug Authority (Currently), Giza 12511, Egypt; 2 Department of Pharmaceutics, College of Pharmacy, Umm Al-Qura University, Makkah 21955, Saudi Arabia; astulbah@uqu.edu.sa; 3 Department of Pharmaceutical Chemistry, College of Pharmacy, King Saud University, Riyadh 11451, Saudi Arabia; 4 Macquarie Medical School, Faculty of Medicine, Health and Human Sciences, Macquarie University, Ryde, NSW 2109, Australia; rania.salama@mq.edu.au; 5 Woolcock Institute of Medical Research, Glebe, NSW 2037, Australia; 6 Department of Pharmaceutical Chemistry, College of Pharmacy, Taif University, P.O. Box 11099, Taif 21944, Saudi Arabia; i.abdelaal@tu.edu.sa; 7 Department of Pharmaceutics, Faculty of Pharmacy, Badr University in Cairo (BUC), Badr City, Cairo 11829, Egypt; 8 Department of Pharmaceutics, Faculty of Pharmacy, University of Sadat City, Menoufia 32897, Egypt; 9 Institute of Pharmaceutical Science, Faculty of Life Sciences & Medicine, King’s College London, London SE1 9NH, UK

**Keywords:** intranasal, ziprasidone, lipid–polymer hybrid, brain targeting, nanocarriers

## Abstract

The feasibility of using lipid–polymer hybrid (LPH) nanocarriers as a potential platform for the intranasal delivery of ziprasidone (ZP), a second-generation antipsychotic, was explored. Different ZP-loaded LPH composed of a PLGA core and cholesterol-lecithin lipid coat were prepared using a single step nano-precipitation self-assembly technique. Modulation of polymer, lipid and drug amounts, as well as stirring-speed-optimized LPH with a particle size of 97.56 ± 4.55 nm and a ZP entrapment efficiency (EE%) of 97.98 ± 1.22%. The brain deposition and pharmacokinetics studies proved the efficiency of LPH to traverse the blood–brain barrier (BBB) following intranasal delivery with a 3.9-fold increase in targeting efficiency compared to the intravenous (IV) ZP solution with a direct nose-to-brain transport percentage (DTP) of 74.68%. The ZP-LPH showed enhanced antipsychotic activity in terms of animals’ hypermobility over an IV drug solution in schizophrenic rats. The obtained results showed that the fabricated LPH was able to improve ZP brain uptake and proved its antipsychotic efficiency.

## 1. Introduction

Despite the great developments in the field of neurology, schizophrenia is a major public health problem worldwide that deteriorates patients’ personal, social, educational and professional functioning [1]. Ziprasidone (ZP) is an antipsychotic benzo-thiazolyl piperazine derivative. ZP is a selective monoaminergic blocker that has an affinity for the H1 histaminergic receptor, serotonin type 2 (5HT2), type 2 dopamine (D2), and type 1 and 2 adrenergic receptors [2,3]. Many neurotherapeutics fail to effectively treat central nervous system (CNS) disorders because they cannot reach the brain [4]. Despite the relatively high brain blood flow, drug delivery to the brain faces many difficulties. Drug delivery is regulated by two physiological barriers, namely, the blood–brain barrier (BBB) and the blood–cerebrospinal-fluid barrier which separate the brain from its blood supply [5]. Intranasal administration (IN) of CNS-acting drugs is a promising route to improve brain targeting [6,7]. Although there was no clear mechanism demonstrating the nose-to-brain delivery pathway, many studies investigated the high nasal mucosa permeability as well as the olfactory and trigeminal nerve pathways, which were the most suggested approaches for nose-to-brain delivery [8,9,10,11]. Furthermore, it is proven that the IN administration of different nanocarriers improves the bioavailability of different CNS drugs by circumventing the BBB and protecting the payloads from degradation [12,13,14,15,16].

Since 2008, lipid–polymer hybrid nanoparticles (LPH) have been developed [17] as nanocarriers. LPHs combine the benefits of both polymer- and lipid-based formulations. The nanocarriers constitute of lipid shells and polymer cores [18,19]. Therefore, LPH, with its features, would combine the advantages of both polymeric and lipid NPs and circumvent their defects [20]. The system’s physical stability and integrity are maintained by the polymeric core. The surrounding lipid shell allows surface functionalization and enhances the system’s cellular absorption while maintaining a high stability in general circulation [21,22]. In addition, LPH overcomes other nanoparticle fabrication-method limitations such as batch-to-batch scale-up variations, structural disintegration with limited circulation time, and content leakage [23,24].

In this study, ZP-loaded LPH-NPs were fabricated with a PLGA core and shell made up of a mixture of lecithin and cholesterol. The formulations were fabricated and statistically optimised. The ability of the LPH system to improve ZP pharmacokinetics and brain uptake following IN delivery was assessed in rats. In addition, the antipsychotic activity of the proposed system was studied in schizophrenic rats.

## 2. Results and Discussion

### 2.1. Preparation and Characterization of Ziprasidone Lipid–Polymer Hybrid (ZP-LPH)

BBD was implemented to investigate the influence of the tested CPPs on the CQAs, namely, particle size (Y1) and EE% (Y2). Twenty-nine formulations were generated by the software and represented in Table 1. The fabricated ZP-LPH formulae had a particle size ranging from 93.75 ± 1.01 to 232.50 ± 3.52 nm, with a PDI of less than 0.25 indicating the formation of monodisperse systems. In addition, the quantified ZP in the proposed LPH was 41.58% ± 1.54 to 95.87% ± 3.11. Based on the highest R^2^ (0.9802 and 0.9730) and the lowest PRESS values (8.39 and 3.86), the quadratic model was selected as the best-fit statistical model for both particle size and EE% responses, respectively (Tables S1 and S2). The ANOVA of the regression coefficients of the obtained responses from BBD for the particle size and EE% of the prepared ZP-LPH and the associated *p*-values (*p* < 0.05) is represented in Tables S3 and S4 respectively.

**Figure 1:**
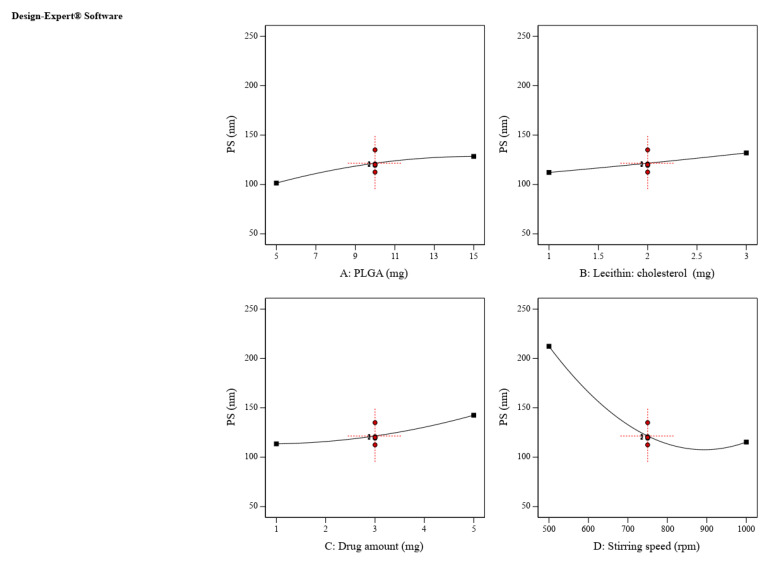
The effect of CPPs on particle size (Y1). Increasing PLGA amount (**A**), lipid amount (**B**) and ZP amount (**C**) had a positive influence on particle size, while an opposite effect was seen with an increasing stirring speed (**D**).

**Figure 2:**
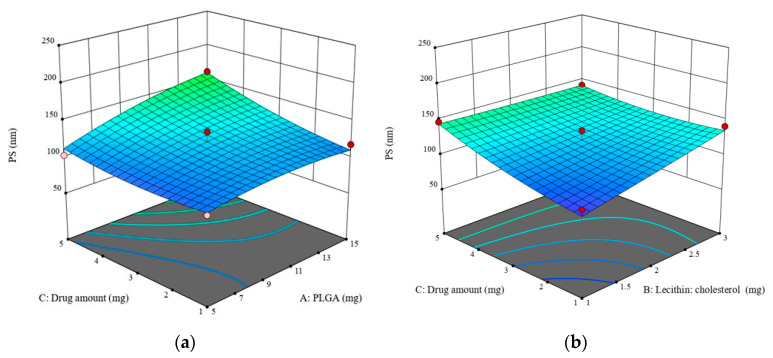
Response 3D plot for the significant parameters’ interactions with ziprasidone LPH particle size (Y1). Interaction of (AC) between PLGA and ZP amounts (**a**). Interaction of (BC) between lipid and ZP amounts (**b**). The positive interaction between the PLGA and ZP amounts indicates an enhanced effect of both variables on LPH particle size. On the contrary, the lipid–drug interaction shows an adverse effect on particle size.

According to Equation (1) and Figure 1, increasing PLGA amount (A), lecithin-cholesterol amount (B) and ZP amount (C) is directly proportional to the increase in particle size, while increasing the stirring speed (C) has a negative effect, i.e., it leads to a decrease in particle size. The positive interaction between PLGA and ZP amounts enhances the combined effect of both variables on LPH particle size, i.e., it increases the particle size. In contrast, the lipid–drug interaction shows an adverse effect on particle size, and leads to the production of larger nano-particulates (Figure 2).

Particle size = 121.5 + 13.5A + 9.87B + 14.5C − 48.5D + 10.31AC − 11.25BC + 42.34D^2^ (1)

This observation could be attributed to the possibility that higher amounts of the organic phase component, PLGA, decrease the evaporation rate, hence producing a larger particle size [25]. Moreover, this viscous solution would oppose the stirring shear force [26] thus producing a larger particle size, as previously reported [25,27,28].

The stirring speed (D), on the other hand, is inversely proportional to particle size. Mechanical shear force produced by increasing the stirring speed resulted in an LPH with smaller particle size [29].

**Figure 3:**
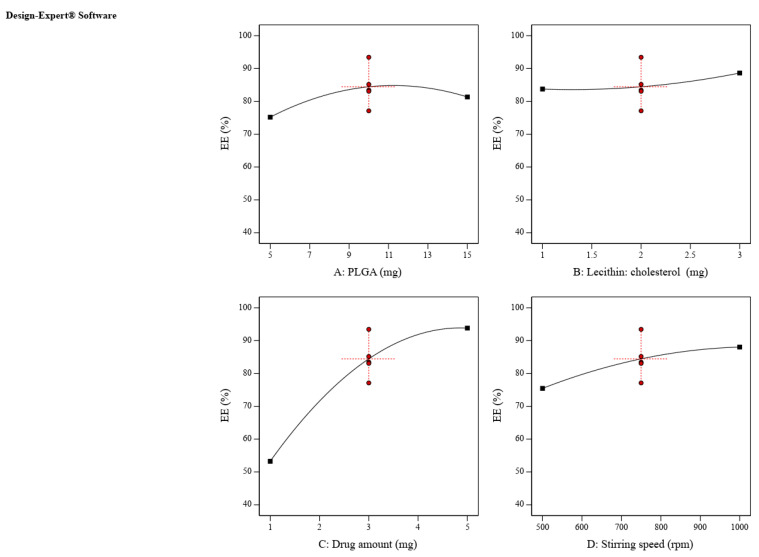
The main effect of the CPPs on EE% (Y2). The PLGA amount (**A**), lipid, drug amount (**B**,**C**) and stirring speed had a positive influence on EE% (**D**).

Equation (2) and Figure 3 represent the effects of the investigated variables on ZP EE%, where a positive correlation between all factors and EE% could be depicted. The larger particle size obtained by increasing PLGA, lecithin-cholesterol and drug amounts could improve the ZP entrapped in the prepared LPH vesicles [30]. Moreover, the barrier effect of the thicker lipid bilayer could have assisted in hindering drug leakage [31]. In addition, cholesterol acts as a stabilizing agent as it can deter the formation of liquid crystal phases that could diminish the drug leakage, and consequently increase the EE% [32].

EE% = 84.44 + 3.07A + 2.43B + 20.28C + 6.29D − 13.75AB − 6.18A^2^ − 10.91C^2^ (2)

The ZP EE% is significantly increased by increasing the stirring speed (D). This could be attributed to the turbulent flow resulting from the high speed applied for a specified period, which could have improved the evaporation of organic solvent, prevented drug leakage, and helped in flash precipitation [33].

**Figure 4:**
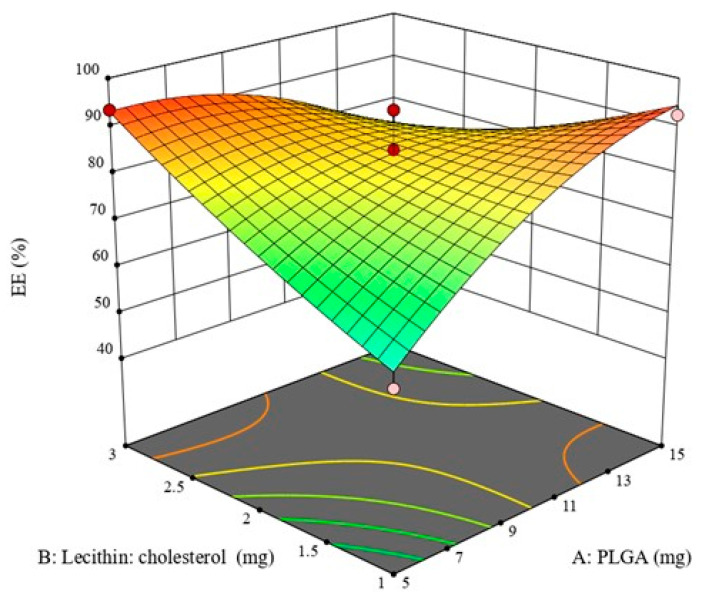
Response 3D plot for the interaction of (AB) between PLGA amount and Lecithin-cholesterol amount on EE% (Y2). The interaction between the polymer and lipid amounts has a negative influence on ZP-LPH EE%.

The interaction between the polymer and lipid amounts had an antagonistic effect on the ZP EE%, as represented in Figure 4. This could be due to the different drug solubilities and polarities in the lipid phase and polymer mixture, which determine the drug EE% [34].

### 2.2. Design Space and Optimization of ZP-LPH

To obtain QTPP, the design space was plotted by superimposing the influence of various CPPs on the contour plots of the CQAs. The optimum CPP values that meet the QTPP criterion are depicted in the yellow area (Figure S1). One ZP-LPH formula was chosen as a checkpoint based on the maximum desirability being equal to 1. The optimized LPH’s composition is shown in Table S5, along with the associated predicted and experimental particle sizes and EE% values. The calculated error % was 4.06 and 1.4% for particle sizes and EE%, respectively, indicating the suitability of the selected model to predict the suitable CPPs to obtain LPH with the anticipated QTPP. The optimized ZP-LPH LE % was 8.57 ± 0.54%, with a negative zeta potential value of −19.68 ± 2.57 (Table 2). The negative charge could be attributed to the lipid coat [35,36,37].

### 2.3. Morphological Structure of the Optimized Selected ZP-LPH

**Figure 5:**
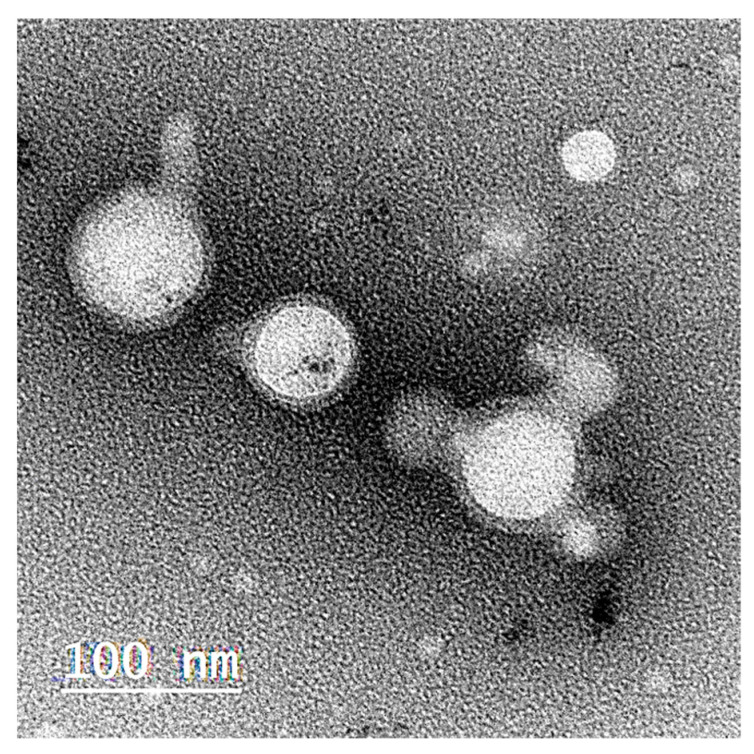
Morphological characterization of the optimized ZP-LPH by a transmission electron microscope. ZP-LPH is a core–shell nanostructure with a particle size in the range of 80–90 nm.

A TEM image of the selected ZP-LPH is illustrated in Figure 5. LPH appeared as a non-aggregated central white PLGA core surrounded by a dark lipid shell coat [38]. The measured ZP-LPH particle size was in the range of 80–90 nm.

### 2.4. In Vitro Drug-Release Study

**Figure 6:**
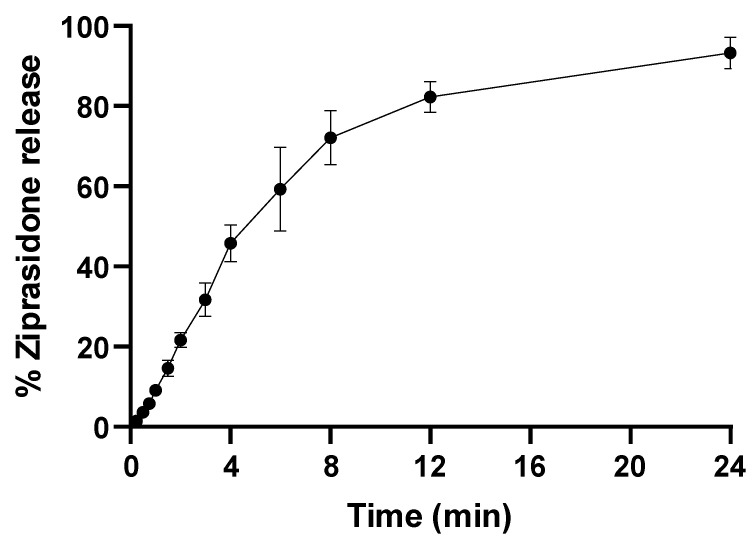
In vitro release profile of drug from ziprasidone LPH. In vitro ZP release from the optimized LPH in simulated nasal fluid pH 6.5. Drug concentration in the dialysate was quantified by HPLC. Data point represents mean and SD (*n* = 3).

By virtue of their features, LPHs combine the advantages of a polymer core engulfing the drug with the added impediment effect of the lipid shell [39]. Figure 6 represents the in vitro ZP release from the optimized LPH in simulated nasal fluid (pH 6.5). The chosen optimized ZP-LPH formula shown in Table 3 showed a biphasic release pattern, where about a 20% burst release in 2 h was followed by a slower release rate up to 24 h. The initial burst release phase was due to the release of ZP from the LPH surface [38], while the slower release phase could be due to the slow partitioning of ZP from the LPH core [16].

### 2.5. In Vitro Cytotoxicity

**Figure 7:**
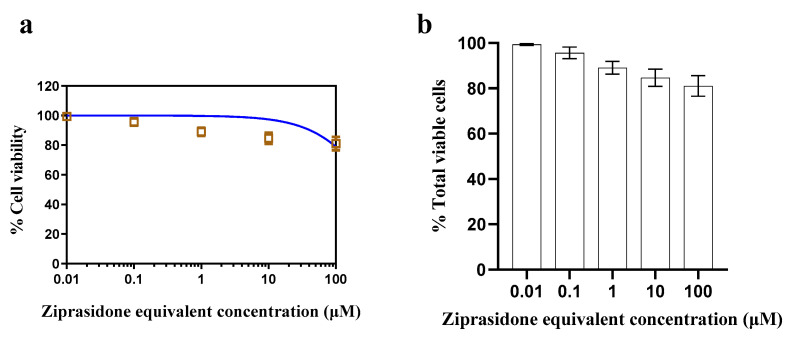
Cell viability assay of ZP-LPH after incubation for 72 h. Calu-3 cells were incubated with ZP-LPH at increasing drug concentrations (0.01–100 μM). Cell viability was assessed by MTT assay and results are presented as % of the viable cells to the untreated cells (**a**,**b**). The optimized LPH showed high cell viability at all tested drug concentrations. Data points are expressed as mean ± SD (n = 5).

ZP-LPH cytotoxicity was examined using Calu-3 cells at increasing doses (0.01 to 100 μM). The optimized LPH, detailed in Table 2, showed high cell viability at all tested drug concentrations after incubation for 72 h (Figure 7). This could be attributed to the biocompatibility of the LPH components. Low toxicity proves the suitability of the selected formula for nasal delivery.

### 2.6. In Vivo Study

**Figure 8:**
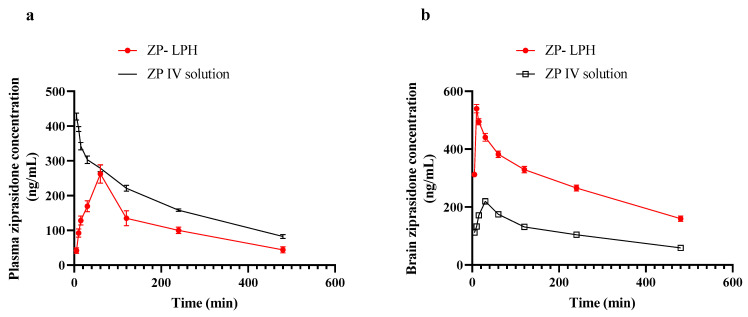
ZP concentrations in rat plasma (**a**) and brain (**b**) after administration of various formulations. Animals received a dose of 2.5 mg/kg of ZP either via IV injection through the tail vein or IN instillation of 10 µL of ZP-LPH in each nostril. At each time point, 6 animals were sacrificed from each group and the concentration of ZP in the plasma and brain was quantified using HPLC. A significantly higher brain ZP concentration was observed at all time points following IN administration of ZP-LPH compared to the IV solution. Data points represent the mean ± SE (n = 6).

Figure 8 shows the plasma and brain concentrations of ZP in rats following intravenous and intranasal injection throughout time, while Table 4 represents the pharmacokinetic parameters in addition to DTE% and DTP. The IN administration of ZP-LPH showed a C_max_ value of 262.23 ± 26.93 ng/mL at 60 min. The calculated AUC_0–480_ min was 881.47 and 1399.76 ng/mL.h for the intranasal ZP-LPH and IV solution, respectively. In addition, both intranasal ZP-LPH and IV solutions had a relatively similar elimination rate from plasma in terms of MRT and K_el_. On the contrary, brain pharmacokinetic data following the IN instillation of ZP-LPH revealed a significantly higher drug concentration at all time points compared with IV administration (*p* < 0.05). In addition, a shorter T_max_ and higher AUC_0–480_ min after IN ZP-LPH administration compared to IV ZP solution indicate the superiority of the IN administration of ZP-LPH over an IV ZP solution (Table 3). The high MRT value indicated the increase in the drug residence at the nasal side. The high DTE% (394.94%) and DTP% (74.68%) values proved the efficiency of the IN administration of LPH in brain targeting [40]. The improved brain pharmacokinetic parameters could be related to the high ZP EE%, the penetration enhancing effect of Tween 80 and the lipophilicity of the lipid coat [15,16,18,41,42]. In addition, lecithin would facilitate the adsorption of apolipoprotein E, which improves transcytosis in a BBB in vitro model through a lysosome-bypassing uptake mechanism [43]. Moreover, it was shown that nanoparticles with a size up to 100 nm could be directly transported to the brain via the olfactory pathway [44,45].

### 2.7. Pharmacodynamic Study

To ensure the improved antipsychotic effect of the proposed nasal ZP-LPH, pharmacodynamics studies of the paw test and open field test were adopted. Schizophrenia-induced rats are characterized by their hyperactivity, which could correspond to the psychomotor agitation present in schizophrenic patients [46]. ZP is reported to prevent hyperlocomotive activity [47,48,49].

#### 2.7.1. Paw Test

**Figure 9:**
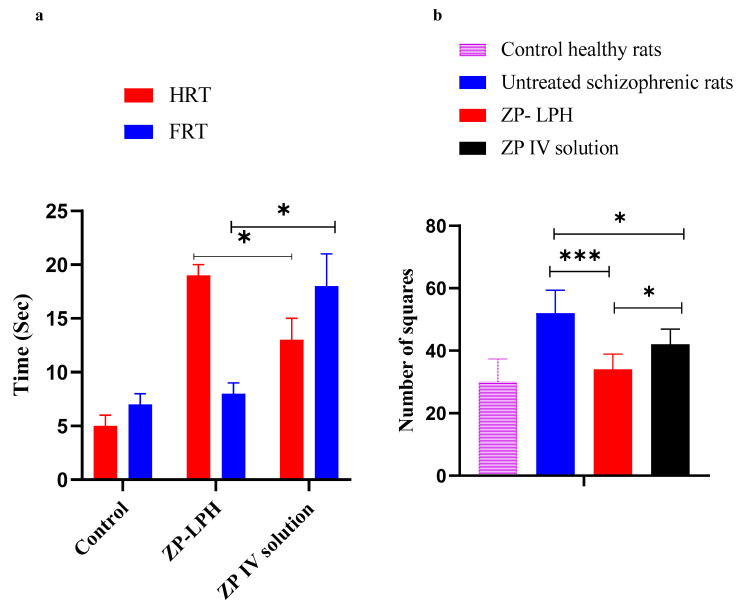
Assessment of pharmacodynamics effect of IV ZP solution and intranasal ZP-LPH in the (**a**) paw test and (**b**) open field test FOR ketamine-induced schizophrenia in rats. Data represent mean ± SE (n = 6). Statistical analysis was performed using ANOVA followed by the Tukey HSD test, * *p* < 0.05.

Forelimb retraction time (FRT) and hindlimb retraction time (HRT) increases were related to the risk of extrapyramidal side effects of the antipsychotic drugs and their potential antipsychotic effects, respectively [50]. As shown in Figure 9A, it was observed that the nasal administration of ZP-LPH resulted in a significantly higher HRT value compared to both the control group and that which received the IV ZP solution injection (*p* < 0.05). The increase in HRT could be correlated with a higher ZP concentration that had reached the brain following IN administration, indicating the brain-targeting effect of the proposed system. On the contrary, the significant reduction in FRT in rats following the administration of IN ZP-LPH compared to the IV solution is an indication of the absence of parkinsonian side effects (*p* < 0.05) [51].

#### 2.7.2. Open Field Test

Figure 9B represents the locomotor activity of schizophrenia-induced rats compared to the control healthy group, expressed as the number of crossed squares in 60 min. Increased locomotor activity is considered a positive schizophrenia symptom [52]. Untreated schizophrenic rats exhibited hyperlocomotion noted by a significant increase in the number of crossed squares compared to animals receiving the IN instillation of ZP-LPH, or healthy rats (*p* < 0.001). In addition, IN administration showed a significant reduction in the crossed-squares number compared to the IV ZP solution (*p* < 0.05). This improvement in antipsychotic activity could be correlated with the selective increase in glutamate and dopamine release in the prefrontal cortex, nucleus accumbens and striatum [53,54]. ZP may inhibit the 5-HT2A receptors on glutamatergic terminals, causing a spontaneous reduction in the stimulating effect of ketamine in the dopamine-glutamate pathway [55,56]. The higher brain ZP concentration was linked to the greater reduction in animal mobility observed with IN ZP-LPH administration compared to the IV ZP solution.

## 3. Materials and Methods

### 3.1. Materials

Ziprasidone (ZP), soya lecithin (LE), cholesterol (CH), dimethylformamide (DMF), dimethylsulfoxide (DMSO), RPMI medium, penicillin, streptomycin, L-glutamine, phosphate-buffered saline (pH 7.4) and foetal bovine serum (FBS) were purchased from Sigma Aldrich (St. Louis, MO, USA). Acetonitrile (HPLC grade), ethanol absolute (HPLC grade) and Tween 80 were obtained from Fluka Chemika-BioChemika, Buchs, Switzerland. Acid terminated DL-lactide/glycolide (50/50) (PLGA intrinsic viscosity = 0.2 dL/g) was generously supplied by Purac Biomaterials (Arkelsedijk 46 P.O. Box 21. Gorinchem, 4206 AC The Netherlands). 

### 3.2. Formulation and Evaluation of Ziprasidone Lipid–Polymer Hybrid (ZP-LPH)

A single-step nano-precipitation self-assembly method was used to formulate different ZP-LPH formulations with a slightly modified methodology from the previously described method by Tahir et al. (2017) [25]. The organic phase was prepared by dissolving different amounts of ZP and PLGA into DMF (1 mL) (Table 4). A 4% *v*/*v* hydroalcoholic solution (9 mL) was prepared at 70°C to dissolve lipid components (lecithin: cholesterol 1:1) and Tween 80 (1% *w*/*v*). For ZP-LPH dispersion preparation, the lipid solution was gradually titrated with the organic phase while being continuously stirred for 2 h at ambient temperature. The ZP-LPH pellets were obtained by centrifugation at 12,000 rpm for 30 min at 4 °C, and then re-dispersed in PBS (pH 7.4).

### 3.3. Box–Behnken Design (BBD) Experimentation and Implementation

BBD was used to design the matrix, examine the response surfaces, and optimize several ZP-LPH formulations (Design-Expert^®^ 13, State-Ease Inc., Minneapolis, MN, USA) [57]. The amount of PLGA (A), lecithin-cholesterol (B) and ZP (C), and the stirring speed (D) were chosen as independent variables (critical process parameters; CPPs) at three different levels (Table 1). Particle size (Y1) and ZP’s entrapment efficiency (EE%) (Y2) were chosen as the critical quality attributes (CQAs). Polynomial equations were created to express the relationship between the CPPs and CQAs. The best-fitting model, either linear or two-factor interactions (2FI), or the quadratic model, was selected based on various statistical indices such as R2 values (adjusted and predicted) and predicted residual error sum of squares (PRESS). The aim of this study was to obtain ZP-LPH with a minimum particle size and maximum EE% as a quality target product profile (QTPP).

### 3.4. Characterization of the Ziprasidone Lipid–Polymer Hybrid (ZP-LPH)

#### 3.4.1. Particle Size, Polydispersity Index (PDI) and Z-Potential Analysis (ZP)

Dynamic light scattering (Nanosizer ZS Series, Malvern Instruments, Southborough, MA, USA) was used to assess particle size and polydispersity index (PDI) and zeta potential [37].

#### 3.4.2. Entrapment Efficiency Percentage (EE%) and Loading Efficiency (LE%)

The entrapment efficiency percentage (EE%) was calculated by quantifying the amount of ZP trapped inside the ZP-LPH pellets. The ZP entrapped amount was obtained by dissolving ZP-LPH pellets in DMF (10 mL) and measuring the ZP amount using a previously validated HPLC method [58].

The HPLC system was composed of (Dionex, Thermo UltiMate 3000 HPLC System), equipped with an LPG-3400SD quaternary pump, a WPS-3000TSL auto sampler, and a variable-wavelength detector (VWD-3000). A reverse-phase C18 column (ACE, 250 × 4.6 mm^2^, 5 μm) was used to separate ZP at 25 °C at 210 nm. The mobile phase consisted of acetonitrile and potassium dihydrogen phosphate buffer (pH 3.6 ± 0.1; 20.0 mM containing 0.2% *v*/*v* triethylamine) at a ratio of 28:72% *v*/*v* and flow rate of 1 mL/min. The calibration curve of ZP in the range of 0.02–3 µg/mL showed a coefficient of determination (R2) of 0.9995 and the limits of detection and quantification were 0.01 and 0.02 µg/mL, respectively. Additionally, the coefficient of variation percentage ranged from 2.6 to 4.75% and the accuracy of determination was 1.8 to 4.3%, with a mean recovery percentage of 96.33 ± 1.35%.

The EE% and LE were determined as follows:

(3) $$\mathrm{EE}\%=\frac{\mathrm{amount}\;\mathrm{of}\;\mathrm{Ziprasidone}\;\mathrm{inside}\;\mathrm{the}\;\mathrm{pelletes}}{\mathrm{Total}\;\mathrm{amount}\;\mathrm{of}\;\mathrm{Ziprasidone}\;\mathrm{added}}*100$$

(4) $$\mathrm{LE}\%=\frac{\mathrm{mass}\;\mathrm{of}\;\mathrm{Ziprasidone}\;\mathrm{inside}\;\mathrm{the}\;\mathrm{pelletes}}{\mathrm{Total}\;\mathrm{mass}\;\mathrm{of}\;\mathrm{Ziprasidone}\;\mathrm{LPH}\;\mathrm{NPs}}*100$$

#### 3.4.3. Transmission Electron Microscopy (TEM)

TEM was used for identifying the selected ZP-LPH nanovesicle morphology (Joel JEM 1230, Tokyo, Japan) as described elsewhere [20]. 

#### 3.4.4. In Vitro Drug Release

The in vitro release of ZP from the optimized LPH formula was assessed using the dialysis method described by Abd-Algaleel et al. with some slight modifications [16]. An aliquot volume of ZP-LPH (equivalent to 5 mg ZP) was placed in the presoaked dialysis membrane with cut off; 10k Da, and 1 mL of simulated nasal fluid of pH 6.5, to mimic nasal mucosa conditions, was added [59]. The membranes were tightly closed and immersed in simulated nasal fluid of pH 6.5 (50 mL) at 37 ± 0.5 °C. The experiment was conducted in a thermostatically controlled shaking water bath at 50 ± 0.1 strokes/min. An aliquot of 0.5 mL was taken out and replaced with preheated release media at different intervals. The released ZP amount was determined using the validated HPLC method [58].

### 3.5. In Vitro Cytotoxicity

An in vitro cytotoxicity study was applied to the optimized ZP-LPH using MTT assay on Calu-3 cells. Calcu-3 cells (American Type Culture Collection, ATCC, Manassas, VA, USA) were cultured in RPMI media supplemented with 10% *v/v* FBS, 50 U/mL penicillin, 50 µg/mL streptomycin and 1% *v/v* L-glutamine and incubated in 5% CO_2_ at 37 °C [60]. Calu-3 cells were seeded in 96-well plates at a density of 7 k/well for 24 h. Cells were incubated with ZP-LPH in the range 0.01–100 μM for 72 h. Subsequently, the incubation media was aspirated and MTT solution (120 μL) was added at 37 °C and 5% CO_2_ for 4 h. The formed formazan crystals were dissolved in 200 μL DMSO then the plate was read at 570 nm using a FLUOstar^®^ Omega plate reader (BMG Labtech) [7].

### 3.6. In Vivo Studies of the Selected Formula

Animal experiments were performed according to the ARRIVE guidelines and approved by the Faculty of Pharmacy, Badr University research ethical committee, (approval number) PT-125-A, Cairo, Egypt. Ninety-six adult male albino rats (aged 4–5 months), weighing about 200 g ± 10% each, were randomly divided into two groups (n = 48), namely, intravenous (IV) ZP solution and IN ZP-LPH. All animals received a dose of 2.5 mg/kg of ZP, either via IV injection through the tail vein or the IN instillation of 10 µL of ZP-LPH in each nostril [7]. Blood samples were collected on heparinized tubes at 5, 10, 15, 30, 60, 120, 240 and 480 min following ZP administration (n = 6 for each time point). Blood samples were centrifuged at 3000 rpm for 30 min at 4 °C (Laborezentrifugen, 2k15; Sigma, Osterode am Harz, Germany). The obtained plasma was stored at −80 °C until analysis. At each time interval, six animals from each group were culled by cervical dislocation and the brain was isolated. Brain tissue was homogenized at 10,000 rpm for 5 min using a tissue homogenizer (Thomas Scintifica, McDonough, GA, USA) [61,62]. Plasma and brain homogenates were deproteinized by mixing them with acetonitrile (1:1 *v*/*v*). The concentration of ZP was quantified using the previously reported HPLC method [58].

The pharmacokinetic parameters in the plasma and brain, as the maximum drug concentration (C_max_), time needed to reach C_max_ (T_max_), area under the concentration-time curve (AUC_0–8_ and AUC_0–∞_), mean residence time (MRT), elimination rate constant (K_el_), and absolute bioavailability, were calculated. In addition, the direct-transport percentage (DTP%) and drug-targeting efficiency percentage (DTE%) were computed as follows: [63].

(5) $$DTE=\frac{({AUC}_{brain}/{AUC}_{plasma})IN}{({AUC}_{brain}/{AUC}_{plasma})IV}*100$$

(6) $$DTP\%=\frac{B_{IN}-B_{X}}{B_{IN}}*100$$

where

$$B_{X}=\frac{B_{IV}}{P_{IV}}*P_{IN}$$

B_IV_ and B_IN_ are the brain AUCs following IV and IN administration, respectively, and P_IV_ and P_IN_ are the plasma AUCs following IV and IN administration, respectively.

### 3.7. Pharmacodynamics Studies

#### 3.7.1. Paw Test

A Perspex platform was constructed with dimensions of 30 × 30 × 20 cm; two small holes (4 cm diameter) for the forelimbs, two large holes (5 cm diameter) for the hindlimbs, and a slit for the tail were located at the top [64]. Eighteen rats were randomly divided into three groups (n = 6 in each) for the control, IN ZP-LPH and IV ZP solution. The rats’ forelimbs were lowered into the holes first, then their hindlimbs, following 30 min of medication administration. Both the forelimb and hind limb retraction times (FRT and HRT) were noted. The FRT measured how long it took for a rat to remove one forelimb, while the HRT measured how long it took to remove one hind limb [65].

#### 3.7.2. Open Field Test in Schizophrenia Rat Model

Eighteen rats were given 25 mg/kg of ketamine intraperitoneally to induce schizophrenia [7]. An open-field device (40 × 40 × 30 cm; Accuscan Instruments, Columbus, OH, USA) divided into 16 small squares was used for the test. Animals were randomized into three groups (n = 6): the control group (no treatment), the IN ZP-LPH group, and the IV ZP solution group. The ZP dose was changed to 2.5 mg/kg/day for one week. Prior to the test, each rat was left in its home cage in the testing area for 2 h before being gently moved by the base of its tail and placed into one of the four corners of the open field, facing the centre, in order to calculate the ambulatory distance of the rat (the total number of squares crossed) [66]. Data were collected over 60 min and were compared to healthy rats (n = 6).

### 3.8. Statistical Analysis

All in vitro experiments were conducted in three replicates, and the recorded results were the mean ± standard deviation (SD). Six replicates were used for in vivo experiments and the data were expressed as mean ± standard error (SE). The student t-test was used to compare two variables. One-way analysis of variance (ANOVA) was used to compare different parameters between groups, followed by the Tukey HSD test. The differences were considered significant at *p <* 0.05.

## 4. Conclusions

In the present study, a suitable LPH platform for the direct nose-to-brain delivery of the antipsychotic drug ziprasidone was successfully fabricated. PLGA, lipid amounts, drug amount and stirring speed were manipulated to achieve LPH with maximum EE% and minimum particle size (<100 nm) to ensure olfactory transport. The IN administration of negatively charged ZP-LPH, with a particle size of 97.56 nm, allowed the efficient brain deposition of ZP more than the IV drug solution. In vivo pharmacokinetics data demonstrated that the IN administration of ZP-LPH significantly increased the AUC of ZP in the brain, with almost a 2.5-fold increase compared to that of the IV ZP solution. Moreover, the study also showed that the brain DTE% and the DTP% of the proposed system were 394.94% and 74.68%, respectively. Pharmacodynamic results proved the ability of IN ZP-LPH to trigger therapeutic efficiency in schizophrenic rats compared to the IV ZP solution. Additionally, diminished extrapyramidal side effects were observed in rats receiving the proposed ZP-LPH over the systemically administrated rats due to the site-specific distribution of the former system.

## Figures and Tables

**Table 1:** Experimental design matrix of the CPPs and the related CQAs.

Run	Critical Process Parameters (CPPs)				Critical Quality Attributes (CQAs)	
	A: PLGA (mg)	B: Lecithin: Cholesterol (mg)	C: Drug Amount (mg)	D: Stirring Speed (rpm)	Particle Size (nm) ^a,c^	EE% ^b,c^
1	10	2	3	750	112.5 ± 3.25	77.11 ± 1.41
2	15	2	5	750	161.25 ± 1.84	94.70 ± 2.45
3	10	2	5	500	232.50 ± 3.52	87.90 ± 3.25
4	10	3	3	1000	123.75 ± 5.11	91.36 ± 1.25
5	10	2	3	750	120 ± 4.11	83.06 ± 3.97
6	10	2	3	750	120.75 ± 3.54	85.16 ± 4.11
7	10	1	3	1000	102.75 ± 1.55	93.46 ± 2.45
8	10	2	3	750	119.25 ± 3.47	83.43 ± 1.95
9	5	2	1	750	100.50 ± 1.97	46.66 ± 2.36
10	15	2	3	500	213.75 ± 4.87	72.72 ± 3.11
11	15	2	1	750	117.75 ± 2.57	47.47 ± 2.45
12	10	3	5	750	141.75 ± 1.79	93.74 ± 2.04
13	10	1	5	750	147 ± 2.33	95.87 ± 3.11
14	5	2	5	750	102.75 ± 2.87	83.06 ± 1.47
15	15	3	3	750	145.5 ± 2.76	73.39 ± 2.63
16	10	2	5	1000	142.5 ± 1.78	93.34± 3.47
17	5	2	3	500	201.75 ± 1.25	68.16 ± 2.01
18	10	2	1	1000	93.75 ± 1.01	57.16 ± 1.98
19	5	3	3	750	102.75 ± 1.14	93.45 ± 1.78
20	5	1	3	750	96 ± 1.01	57.44 ± 1.25
21	5	2	3	1000	103.5 ± 2.21	78.58 ± 2.11
22	15	2	3	1000	126.75 ± 1.36	83.61 ± 2.05
23	15	1	3	750	104.25 ± 1.65	92.40 ± 1.47
24	10	3	3	500	221.25 ± 1.07	77.91 ± 1.39
25	10	2	1	500	199.5 ± 2.16	41.58 ± 1.54
26	10	3	1	750	141 ± 2.11	62.32 ± 1.20
27	10	1	3	500	206.25 ± 2.09	73.73 ± 1.21
28	10	2	3	750	135 ± 1.87	93.43 ± 0.99
29	10	1	1	750	101.25 ± 1.62	49.99 ± 1.01

^a^ Particle size was measured by DLS; ^b^ Calculated as percentage of initial ziprasidone added, determined directly by HPLC; ^c^ Expressed as mean ± SD (n = 3).

**Table 2:** In vitro characterization of the optimized ZP-LPH.

PLGA Amount (mg)	Lecithin: Cholesterol Amount (mg)	ZP Amount (mg)	Stirring Speed (rpm)	Particle Size (nm) ^a,e^	PDI ^a,c^	Zeta Potential (mV) ^b,e^	EE% ^c,e^	LE% ^d,e^
5.7	3	3.4	850	97.56 ± 4.55	0.128 ± 0.018	−19.68 ± 2.57	97.98 ± 1.22	8.57 ± 0.54

^a^ Particle size was measured by DLS; ^b^ Zeta potential was determined by electrophoresis; ^c^ Calculated as percentage of initial ziprasidone added, determined directly by HPLC; ^d^ Calculated as percentage of entrapped ziprasidone weight to the total LPH weight; ^e^ Expressed as mean ± SD (n = 3).

**Table 3:** Pharmacokinetic parameters of IV ZP solution and IN ZP-LPH ^a,b^.

Parameter	Plasma		Brain	
	Ziprasidone IN LPH	Ziprasidone IV Solution	Ziprasidone IN LPH	Ziprasidone IV Solution
C_max_ (ng/mL)	262.23± 26.93	--------	539.96± 14.87 *	219.64 ± 9.56
T_max_ (min)	60	--------	10	30
AUC_0–480 min_ (ng/mL.h)	881.47 ± 25.63	1399.76 ± 24.32 *	2202.56 ± 50.97 *	885.59 ± 45.78
AUC_0–∞_(ng/mL.h)	1128.28 ± 39.68	1859.62 ± 19.65 *	3370.63 ± 41.23 *	1266.75 ± 33.25
MRT (h)	4 ± 0.52	4.2 ± 0.47	4.88 ± 0.24	4.59 ± 0.35
K_el_ (h^−1^)	0.18 ± 0.01	0.17 ± 0.02	0.13 ± 0.014 *	0.15 ± 0.013
Absolute bioavailability (F%)	62.97	100	--------	--------
DTE (%)	--------	--------	394.94	--------
DTP (%)	--------	--------	74.68	--------

^a^ All data are expressed as mean ± SE (n = 6); ^b^ Statistical analysis was performed using student *t*-test, * *p* < 0.05.

**Table 4:** Critical process parameter levels, quality attributes and quality target product profiles of ziprasidone LPH preparation using BBD.

Critical Process Parameters (Coded Independent Variables)	Levels		
	Low (−1)	Medium 0	High (1)
A: PLGA amount (mg)	5	10	15
B: Lecithin: Cholesterol amount (mg)	1	2	3
C: Drug amount (mg)	1	3	5
D: Stirring speed (rpm)	500	750	1000
Critical Quality attributes Quality target product profile (Responses) (Constraints)			
1: Particle size (nm) Y2: Entrapment efficiency EE (%)	Minimum Maximize		

## Data Availability

Data is contained within the article and Supplementary Material.

## Supplementary Materials

The following supporting information can be downloaded at: https://www.mdpi.com/article/10.3390/ph16060886/s1, Figure S1: Overlay plots depicting the design space region for the ziprasidone LPH. The; Table S1: Model summary statistics for particle size (Y1); Table S2: Model summary statistics for ziprasidone LPH EE% (Y2); Table S3: ANOVA of the obtained data from BBD for the particle size of ziprasidone LPH and associated *p*-values; Table S4: ANOVA of the obtained data from BBD for the EE% of ziprasidone LPH and associated *p*-values; Table S5: The experimental and predicted particle size and EE % of the optimized ziprasidone LPH.
