# Supplementary material for: Evaluation of Brain Targeting and Antipsychotic Activity of Nasally Administrated Ziprasidone Lipid–Polymer Hybrid Nanocarriers
Source: Pharmaceuticals (Basel). 2023 Jun 15;16(6):886. doi: 10.3390/ph16060886 (PMC10301809; doi:10.3390/ph16060886)
Supplement: Supplementary file 1 [file pharmaceuticals-16-00886-s001.zip › pharmaceuticals-2433418-supplementary.pdf]

Supplementary data

Table S1. Model summary statistics for particle size (Y1).

Source	Std. Dev.	R^2	Adjusted R^2	Predicted R^2	PRESS	
Linear	25.48	0.6864	0.6341	0.5269	23507.76	
2FI	28.14	0.7132	0.5539	0.1312	43171.24	
**Quadratic[a]**	**8.39**	**0.9802**	**0.9604**	**0.9087**	**4534.65**	**Suggested**
Cubic	9.18	0.9898	0.9525	0.3128	34146.63	Aliased

[a] Adequate precision equals 22.71 and coefficient of variation (C.V.) % is 6.02.

Table S2. Model summary statistics for ziprasidone LPH EE% (Y2).

Source	Std. Dev.	R^2	Adjusted R^2	Predicted R^2	PRESS	
Linear	9.37	0.7264	0.6808	0.5980	3098.08	
2FI	8.28	0.8398	0.7508	0.5897	3162.32	
**Quadratic**	**3.86**	**0.9730**	**0.9459**	**0.9195**	**620.35**	**Suggested**
Cubic	4.95	0.9809	0.9109	0.8028	1519.43	Aliased

Table S3. ANOVA of the obtained data from BBD for the particle size of ziprasidone LPH and associated p-values.

Source	Sum of Squares	Df [a]	Mean Square	F-value	p-value [b]
**Model**	48704.62	14	3478.90	49.45	< 0.0001
A-PLGA	2187.00	1	2187.00	31.09	< 0.0001
B-Lecithin: cholesterol	1170.19	1	1170.19	16.63	0.0011
C-Drug amount	2523.00	1	2523.00	35.87	< 0.0001
D-Stirring speed	28227.00	1	28227.00	401.26	< 0.0001
AB	297.56	1	297.56	4.23	0.0589
AC	425.39	1	425.39	6.05	0.0276
AD	31.64	1	31.64	0.4498	0.5133
BC	506.25	1	506.25	7.20	0.0179
BD	9.00	1	9.00	0.1279	0.7259
CD	62.02	1	62.02	0.8816	0.3637
A^2	282.02	1	282.02	4.01	0.0650
B^2	1.83	1	1.83	0.0260	0.8741
C^2	276.70	1	276.70	3.93	0.0673
D^2	11630.23	1	11630.23	165.33	< 0.0001
**Residual**	984.84	14	70.35		
Lack of Fit	713.72	10	71.37	1.05	0.5250
Pure Error	271.12	4	67.78		
**Cor Total**	49689.47	28			

[a] Degree of freedom
[b] *$p < 0.05$

Table S4. ANOVA of the obtained data from BBD for the EE% of ziprasidone LPH and associated p-values.

Source	Sum of Squares	df	Mean Square	F-value	p-value
**Model**	7498.68	14	535.62	36.00	< 0.0001
A-PLGA	113.72	1	113.72	7.64	0.0152
B-Lecithin: cholesterol	71.38	1	71.38	4.80	0.0459
C-Drug amount	4938.04	1	4938.04	331.89	< 0.0001
D-Stirring speed	475.25	1	475.25	31.94	< 0.0001
AB	756.52	1	756.52	50.85	< 0.0001
AC	29.34	1	29.34	1.97	0.1820
AD	0.0566	1	0.0566	0.0038	0.9517
BC	52.34	1	52.34	3.52	0.0817
BD	9.83	1	9.83	0.6607	0.4299
CD	25.70	1	25.70	1.73	0.2099
A^2	247.95	1	247.95	16.66	0.0011
B^2	19.77	1	19.77	1.33	0.2683
C^2	772.31	1	772.31	51.91	< 0.0001
D^2	47.27	1	47.27	3.18	0.0964
**Residual**	208.30	14	14.88		
Lack of Fit	70.25	10	7.03	0.2036	0.9812
Pure Error	138.05	4	34.51		
**Cor Total**	7706.99	28			

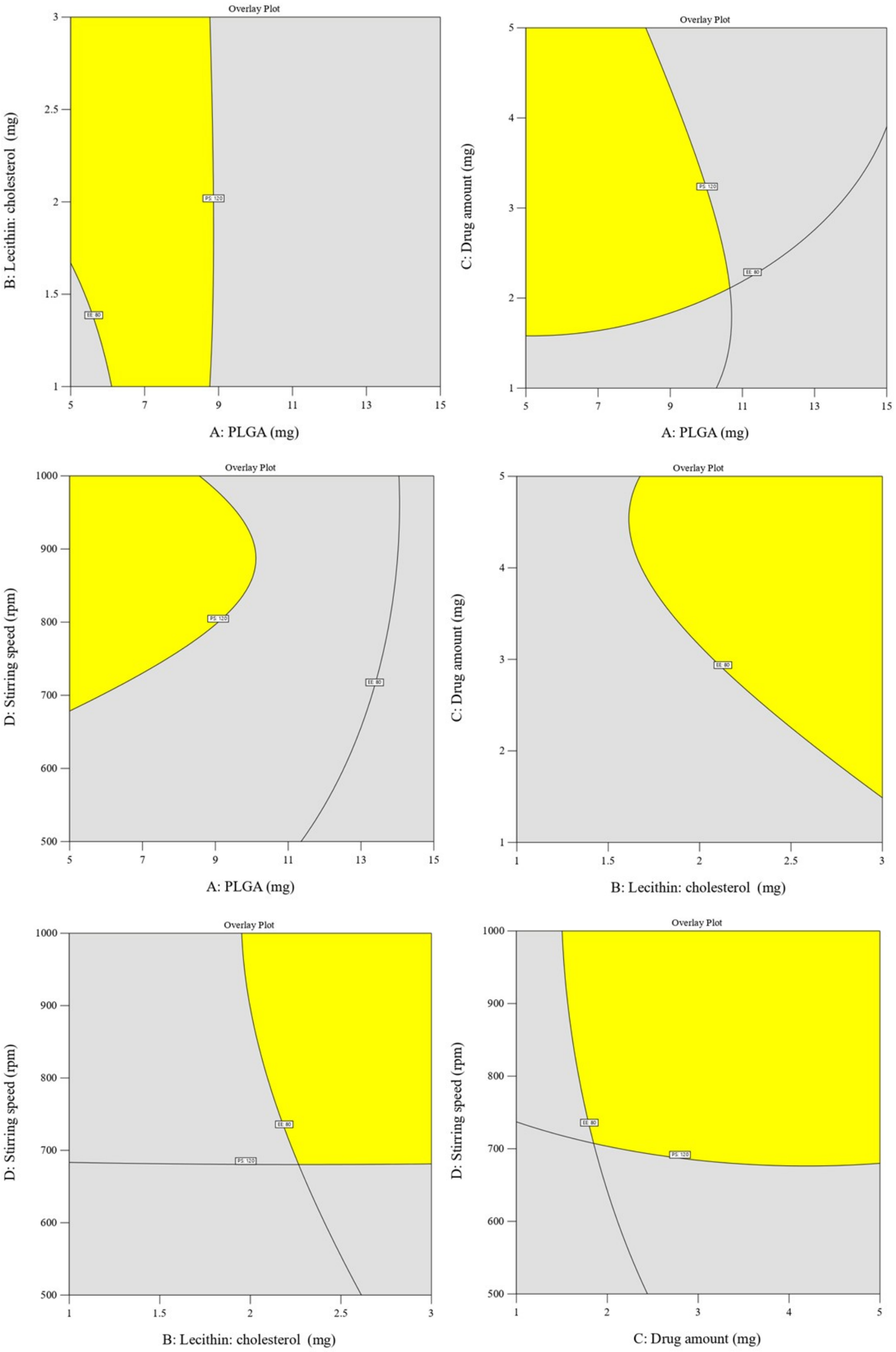

Figure S1. Overlay plots depicting the design space region for the ziprasidone LPH. The design space was plotted by overlapping different CPPs influence on CQAs contour plots to obtain QTPP. The yellow area represents the values of CPPs when optimized to fulfill QTPP criteria; minimum particle size and maximum EE%.

Table S5. The experimental and predicted particle size and EE % of the optimized ziprasidone LPH.

Parameter	PLGA amount (mg)	Lecithin: cholesterol amount (mg)	Ziprasidone amount (mg)	Stirring speed (rpm)	Experimental	Predicted	% Pre. error
Particle size (nm)[a,c]	5.7	3	3.4	850	97.56±4.55	93.59	4.06
EE %[b, c]					97.98±1.22	96.6	1.4

[a] Particle size was measured by DLS.
[b] Calculated as percentage of initial ziprasidone added, determined directly by HPLC.
[c] Expressed as mean ± SD (n=3).
